# Factors affecting stability of plasma brain-derived neurotrophic factor

**DOI:** 10.1038/s41598-020-77046-6

**Published:** 2020-11-19

**Authors:** Jocelyn M. Wessels, Ravi K. Agarwal, Aamer Somani, Chris P. Verschoor, Sanjay K. Agarwal, Warren G. Foster

**Affiliations:** 1 Department of Obstetrics and Gynecology, HSC-3N52D, McMaster University, 1280 Main Street, West, Hamilton, ON L8S 4K1 Canada; 2 Department of Obstetrics and Gynecology and Reproductive Sciences, Center for Endometriosis, Research and Treatment, University of California, San Diego, La Jolla, CA USA; 3 Health Sciences North Research Institute, Sudbury, ON Canada

**Keywords:** Biochemistry, Biomarkers

## Abstract

Circulating concentrations of brain-derived neurotrophic factor (BDNF) have been linked to cancer, neuropsychiatric, diabetes, and gynecological disorders. However, factors influencing plasma storage and subsequent BDNF quantification are incompletely understood. Therefore, the anticoagulant used in plasma separator tubes, storage-time, storage-temperature, and repeated freeze–thaw cycles on circulating BDNF concentrations was evaluated. Peripheral blood samples were collected from healthy women (n = 14) and men (n = 10) recruited prospectively from McMaster University (August 2014). Blood was collected from the cubital vein into plasma separator tubes containing five different anticoagulant systems [K2EDTA, Li-Hep, Li-Hep (gel), Na-Hep, Na-Hep (glass)], and placed on ice for transport to the lab for centrifugation. Plasma samples (n = 16) collected in K2EDTA tubes from women recruited to a previous study (April 2011 to December 2012) were used to determine the effect of multiple freeze–thaw cycles. Plasma BDNF was quantified using a commercially available ELISA kit. Plasma concentrations of BDNF were significantly affected by the type of plasma separator tube, storage-time, and number of freeze–thaw cycles. Storage temperature (− 20 vs. − 80 °C) did not significantly affect the quantity of BDNF measured as mean BDNF concentrations generally fell within our calculated acceptable change limit up to 6 months in the freezer. Our results suggest that for quantification of circulating BDNF blood collected in K2EDTA tubes and plasma stored up to 6 months at either − 20 or − 80 °C produces reproducible results that fall within an acceptable range. However, plasma samples stored beyond 6 months and repeated freeze–thaw cycles should be avoided.

## Introduction

Brain derived neurotrophic factor (BDNF) is a member of the nerve growth factor (NGF) family, a group of soluble polypeptides best known for their role in neurogenesis, differentiation, neurite survival, and synaptic plasticity^1–3^. The clinical utility of circulating concentrations of BDNF have been explored in neuropsychiatric disorders such as obsessive compulsive disorder^4^, eating disorders^5^, depression^6–9^, schizophrenia^10^, bipolar disorder^9,11,12^, as well as neurodegenerative diseases including Parkinson’s, Huntington’s, and Alzheimer’s disease^13,14^. Furthermore, increased circulating BDNF concentrations relative to controls have been linked with several different cancers^15–17^ and gynecological disorders including polycystic ovarian syndrome^18^ and endometriosis^19–21^. Indeed, women with endometriosis have elevated circulating BDNF concentrations compared to women without disease^19–21^, which correlated with pelvic pain^21^, and decreased after surgical removal of lesions^19^. However the clinical value of BDNF as a marker of endometriosis has also been challenged^21,22^. While some studies find BDNF to be a useful clinical marker of endometriosis or certain endometriotic lesion types^19–21^, others find the difference in BDNF between women with and without endometriosis is not predictive of disease^21,22^. The reasons for variable and divergent findings between studies is difficult to resolve, but could include the fact that some studies quantified BDNF in plasma whilst others used serum, or that different ELISA kits were used for quantification. Furthermore, the clotting time and temperature during sample processing have previously been shown to influence measures of circulating BDNF^23–25^. Thus, we postulate that the lack of standardized blood collection and sample handling protocols contribute to variability in measured concentrations of BDNF, which may explain discrepancies in the literature and impact clinical interpretation.

BDNF circulates free in peripheral blood, but is also stored in platelets^26–28^. As platelets are a major source of BDNF, the quantity of BDNF measured in peripheral blood is affected by clotting and platelet activation. This presents a challenge for quantifying free, circulating BDNF as a clinical marker in the peripheral blood because platelet-derived BDNF is a possible confounder that few studies quantifying BDNF have considered^29^. Time from sample collection to processing and temperature during processing lead to higher BDNF concentrations^23–25^. In addition, several subject specific factors are known to affect circulating concentration of BDNF including: age, body weight^30^, stress^31^, and exercise^32^. Study subject sex was also associated with lower platelet concentrations of BDNF in females compared to males^30^ and the plasma concentration is influenced by menstrual cycle stage^33^. Moreover, BDNF expression and circulating concentrations are influenced by gonadal steroids^34^ with lower concentrations measured in the luteal phase compared to the follicular phase. Circulating concentrations have also been shown to follow a circadian rhythm^35^. However, the effect of the type of anticoagulant used during plasma collection, sample handling, processing, storage, and repeated freeze–thaw cycles on BDNF concentration are ill defined, rendering comparison of BDNF values between studies difficult. Therefore, the objective of this study was to evaluate the effect of anticoagulant routinely used in commercial plasma collection tubes, freezer storage-temperature, storage-time, and repeated freeze–thaw cycles on plasma concentrations of BDNF.

## Methods

### Study participants

This study was approved by the Research Ethics Board, McMaster University (REB#06-064, 14-066-T), and all participants provided written informed consent prior to sample collection, and completed our basic study questionnaire probing demographics, medication use, smoking status, and date of last menstruation (for females). The menstrual cycle phase on the day of blood collection was estimated in the female participants using their date of last menstruation, and assuming a regular 28 day cycle.

Volunteer study participants, women (n = 14) and men (n = 10), were prospectively recruited on one day in August 2014 from McMaster University (REB#14-066-T). In addition, plasma samples collected between April 2011 and December 2012 in K2EDTA tubes from women (n = 16) in a prior study^20^ according to our approved ethics protocol (REB#06-064) were available to determine the effect of multiple freeze–thaw cycles on BDNF concentration, as described below. The study and all procedures were carried out in accordance with relevant guidelines and relevant Governmental regulations governing human research.

### Blood collection, plasma isolation and storage

Peripheral blood was collected from the cubital vein of non-fasting study participants into 5 plasma separator tubes (BD Canada, Mississauga, ON, Canada) by a nurse at McMaster University Medical Centre over a two hour period (11 am–1 pm). Plasma separator tubes used (Table 1) included potassium EDTA (K2EDTA), lithium heparin (Li-Hep), lithium heparin gel separator (Li-Hep (gel)), sodium heparin plastic (Na-Hep), and sodium heparin glass (Na-Hep (glass)).

**Table 1:** Vacutainer tube material, anticoagulant system and catalogue number.

Tube name	Tube material	Anticoagulant	Top colour	Contains gel?	BD catalog #
K2EDTA	Plastic	Potassium EDTA	Purple	No	366,643
Li-Hep	Plastic	Lithium heparin	Green	No	367,880
Li-Hep (gel)	Plastic	Lithium heparin	Light green	Yes	367,962
Na-Hep	Plastic	Sodium heparin	Green	No	367,874
Na-Hep (glass)	Glass	Sodium heparin	Green	No	366,480

**Figure 1:**
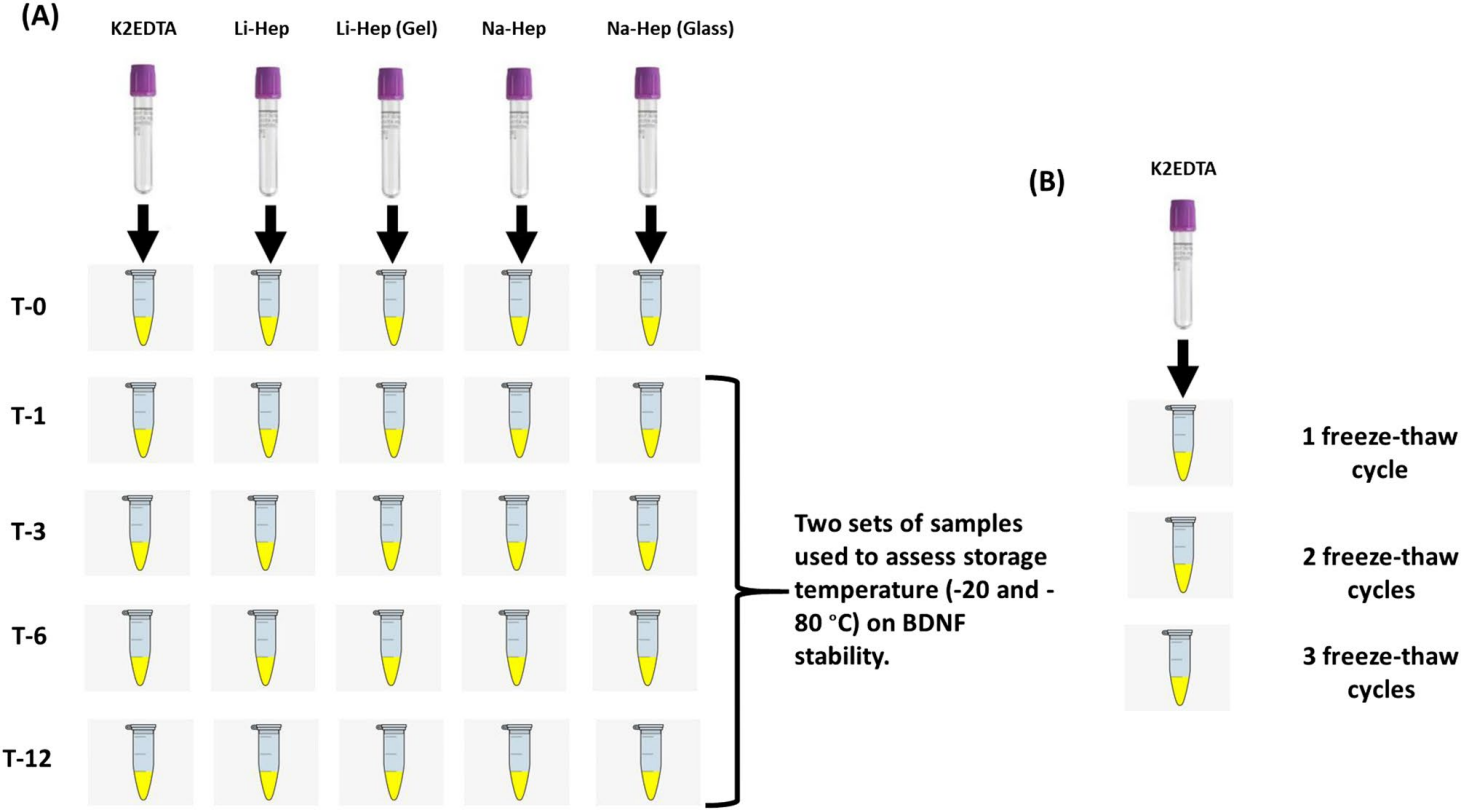
Experimental design flow diagram. (**A**) In the first experiment, to assess the effect of the type of anticoagulant system and storage-temperature on BDNF stability, peripheral blood was collected from the cubital vein by venipuncture into five plasma collection tubes (K2EDTA, Li-Hep, Li-Hep (gel), Na-Hep, and Na-Hep (glass). For each participant (14 female and 10 males), approximately 200 µl of plasma from each of the five plasma collection tubes was aliquoted into 1.8 mL cryovials. One 200 µl aliquot of plasma from each of the five plasma collection tubes was refrigerated at 4 °C overnight, and used to quantify BDNF the following day (Time 0; never frozen). Four aliquots per collection tube were frozen at − 20 and four were frozen at − 80 °C until required for analysis at 1, 3, 6, and 12 months post-collection. (**B**) To evaluate the effect of repeated freeze–thaw cycles (1–3 cycles) on BDNF stability, plasma samples (n = 16) collected in K2EDTA tubes from women recruited to a previous study were used for subsequent BDNF analysis. The first freeze–thaw to quantify BDNF by ELISA occurred within 6 months of freezing and subsequent freeze-thaws (#2 and 3) occurred randomly within the next 6 months. K2EDTA = potassium EDTA, Li-Hep = lithium heparin, and Na-Hep = sodium heparin.

Blood collection tubes were inverted approximately five times to mix, placed on ice, transferred to the laboratory, and placed at 4 °C until they were centrifuged at 3,000 rpm for 30 min to isolate plasma (Fig. 1). All samples were processed in back-to-back batches by the same technician. The total time to isolate all of the plasma (120 separator tubes) was 4 h; whole blood was maintained at 4 °C throughout. For each participant, approximately 200 µl of plasma from each of the 5 plasma collection tubes (K2EDTA, Li-Hep, Li-Hep (gel), Na-Hep, and Na-Hep (glass)) was aliquoted into 1.8 mL cryovials (Sarstedt, Montreal, QC, Canada). Four aliquots/collection tube were frozen at − 20 °C and 4 were frozen at − 80 °C until required for analysis at 1, 3, 6, and 12 months post-collection. One 200 µl aliquot of plasma from each of the 5 plasma collection tubes was refrigerated at 4 °C overnight, and used to quantify BDNF the following day (Time 0; never frozen).

### BDNF freeze–thaw

The effect of repeated freeze–thaw cycles on plasma BDNF concentration was assessed using aliquots of plasma isolated between April 2011 and December 2012 using K2EDTA plasma separator tubes obtained during our prior study^16^. This plasma had been isolated within 30 min of blood collection by centrifuging at 3,000 rpm for 30 min (as above). It was subsequently aliquoted and frozen at − 80 °C. The first freeze–thaw to quantify BDNF by ELISA occurred within 6 months of freezing. Subsequent freeze-thaws (#2 and 3) occurred randomly within the next 6 months. In total, these plasma aliquots were thawed 3 times between the time they were initially collected and 1 year following collection.

### BDNF assay

BDNF concentrations were quantified in duplicate using the BDNF Emax immunoassay ELISA (Promega, Madison, WI, USA) in fresh (Time 0) or frozen (Time 1, 3, 6, and 12 months) plasma following the manufacturer’s protocol. This kit has been reported to quantify both mature and pro-BDNF^36^. All samples for each time point were analyzed together to minimize variation from the use of assay kits from different lot numbers. Frozen plasma was thawed at room temperature prior to quantification. Briefly, 96 well NUNC maxisorp plates (Fisher Scientific, Ottawa, ON, Canada) were coated with anti-human BDNF antibody overnight. Plasma samples were diluted 1:7 with the provided sample buffer. Following incubation, the absorbance was read at 450 nm within 30 min using a Biotek Synergy spectrophotometer (Fisher Scientific). Intra-assay coefficient of variation (CV) was reported to be 2.9% in the manufacturer’s protocol. The inter-assay CV was calculated using the kit standards on all (n = 27) BDNF ELISA plates used. The inter-assay CV was 17.3%. We calculated an acceptable change limit to estimate the expected mean percentage variation range in plasma BDNF concentrations as previously described^37^. Briefly, the inter-assay CV (17.3%) was multiplied by 2.77 which was derived from Z√2 where Z = 1.96 and determined by the 95% confidence interval value for bi-directional changes. From this number, the expected range of the means for each plasma separator was calculated to be ± 47.9% of the mean concentration of BDNF at Time 0 (the most accurate measure of BDNF in each plasma separator tube). Thus, if there is no effect of storage (time or temperature), the mean concentration of BDNF recovered from stored plasma should fall within ± 47.9% of the individual Time 0 mean for each separator tube.

### Data and statistical analysis

Data were tested for normality and statistical analyses performed using GraphPad Prism (GraphPad Software Inc., La Jolla, CA). The effect of type of plasma separator tube on BDNF concentration was stratified by sex and assessed in females by Kruskal–Wallis test and Dunn’s post-hoc test, and in males by one-way ANOVA and Tukey’s post-hoc test. Data was also analyzed for both sexes combined by one-way ANOVA and Tukey’s post-hoc test. We used a linear mixed effects model, accounting for repeated measures within participant, to assess the effect of covariates (participant age, sex, and menstrual cycle stage) on circulating BDNF concentrations across tube type and storage time and temperature, as these factors have previously been shown to influence BDNF^30,33^. Two models were fit, both including tube type, storage time and storage temperature: one was performed on all participants and was additionally adjusted for age and sex (Supplemental Fig. 1A) and the other was performed only on female participants, and was additionally adjusted for age and menstrual cycle stage (Supplemental Fig. 1B). Results are presented as the regression coefficient and 95% confidence interval; for age, the coefficient is relative to a 1-year change, and for categorical variables, the coefficient is relative to the reference category: ie. sex = female, cycle stage = hormonal contraceptives (vs. menstrual, proliferative, secretory), tube = K2EDTA, time = 0, and temperature =  − 20 °C. Mixed models were performed in the R environment.

The effect of time stored at − 20 °C or − 80 °C on BDNF concentration in plasma isolated from the 5 plasma collection tubes (K2EDTA, Li-Hep, Li-Hep (gel), Na-Hep, and Na-Hep (glass)) was assessed in males and females combined by Kruskal–Wallis tests and Dunn’s post hoc tests. The concentration of BDNF recovered from plasma stored at − 20 versus − 80 °C was statistically compared in males and females combined by t-test (for normally distributed data) and Mann–Whitney U test (for non-normally distributed data) following 1, 3, 6, or 12 months in the freezer. The effect of the number of freeze–thaw cycles on BDNF concentration in plasma collected from K2EDTA tubes was assessed by one-way ANOVA with Tukey’s post hoc test.

Most data are presented as box plots with the edges of the box plot representing the 25th and 75th percentiles, while the line through the box represents the median (50%), and the plus sign (+) indicates the mean. Whiskers on the boxes represent the 5th and 95th percentiles. A *p* value ≤ 0.05 was considered significant. All data analyzed in this manuscript can be found in Supplemental Table 1.

### Ethical approval and consent to participate

This study was approved by the Research Ethics Board, McMaster University (REB#06-064, 14-066-T), and all participants provided written informed consent prior to entry inot the study and sample collection.

## Results

### Study participants

The mean (± SD) age of study participants was 24.7 ± 3.9 for women and 32.0 ± 18.6 years for men (Table 2). Of the female participants 57% were Caucasian and 43% were Asian whereas the males were equally split between Caucasian and Asians. The majority of females identified as students whilst males were split equally between employed and students. All study participants were non-smokers, and 43% of females were currently using prescription medications (including oral contraceptives), whilst 20% of males were using prescription medications. Menstrual cycle phase was estimated for female participants using their date of last menstruation, and assuming a regular 28 day cycle. The majority of the women were in the secretory phase of the menstrual cycle (43%), while 14% were in the proliferative phase and 7% were in the menstrual phase. Additionally, 36% of women were currently using oral contraceptives.

**Table 2:** Participant demographics.

Characteristic	Female	Male
Number of participants	14	10
Mean age, years (SD)	24.7 (3.9)	32.0 (18.6)
**Ethnicity, N (%)**		
Caucasian	8 (57%)	5 (50%)
Asian	6 (43%)	5 (50%)
**Occupational status, N (%)**		
Employed	4 (29%)	5 (50%)
Student	10 (71%)	5 (50%)
**Smoking status, N (%)**		
Non-smoker	14 (100%)	10 (100%)
**Current prescription medication use, N (%)**		
Yes	6 (43%)	2 (20%)
No	8 (57%)	8 (80%)
**Menstrual cycle phase, N (%)**		
Menses	1 (7%)	–
Proliferative	2 (14%)	–
Secretory	6 (43%)	–
Oral contraceptives	5 (36%)	–

In the freeze–thaw experiment, plasma samples (n = 16) were selected from samples collected as part of a prior study in our laboratory^20^. The mean age of women whose samples were used was 34.3 ± 6.1 years of which 88% identified as Caucasian, 6% as Asian, and 6% as other. The plasma samples were selected to represent a broad range of BDNF concentrations (169.5–1126.15 pg/mL) at the first thaw after storage at − 80 °C, which occurred within 6 months of sample collection.

### Plasma separator tube impacts BDNF quantification

**Figure 2:**
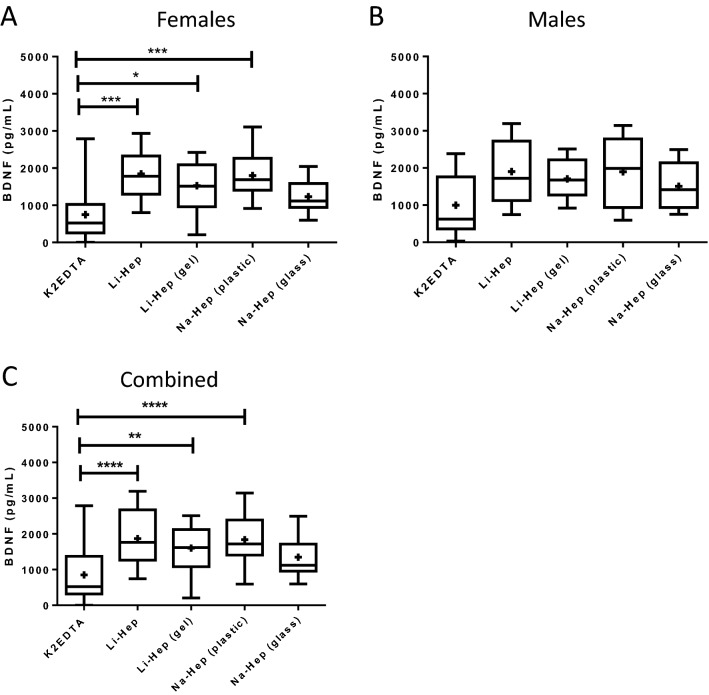
Plasma separator tube impacts BDNF quantification. Peripheral blood was collected into 5 plasma separator tubes per participant and BDNF was quantified by ELISA on fresh plasma samples (Time 0). (**A**) Plasma samples were stratified by sex, and in females, the plasma collected using the K2EDTA separator had significantly lower quantities of BDNF than the lithium heparin, lithium heparin with gel, and sodium heparin tubes (*p* = 0.0001, one-way ANOVA with Tukey’s post hoc), while (**B**) no significant difference in the concentration of plasma BDNF was observed between types of tubes in males. BDNF: brain-derived neurotrophic factor. (**C**) Plasma collected in K2EDTA tubes had significantly less BDNF than the lithium heparin, lithium heparin with gel, and sodium heparin tubes (*p* ≤ 0.0001, one-way ANOVA with Tukey’s post hoc), while the amount of BDNF recovered in the sodium heparin (glass) was comparable to the K2EDTA tubes. K2EDTA: potassium EDTA. Li-Hep: lithium heparin. Na-Hep: sodium heparin. *p ≤ 0.05, **p ≤ 0.01, ***p ≤ 0.001, ****p ≤ 0.0001. + : indicates the mean.

In order to discern if there was an effect of sex on plasma BDNF concentration recovered from each of the plasma separator tubes, the plasma samples from the present study were stratified by sex (Fig. 2A,B). In females, the plasma collected using the K2EDTA separator had significantly lower quantities of BDNF than the Li-Hep, Li-Hep (gel), and Na-Hep separators, (747.7 ± 193.7 vs. 1844.0 ± 177.0, 1526.0 ± 174.5, 1797.0 ± 152.6 pg/mL; *p* = 0.0001, Kruskal–Wallis test with Dunn’s post hoc), while no significant difference in the concentration of plasma BDNF was observed between types of anticoagulant used in the plasma separator tubes in males. Overall, when samples from males and females were combined, plasma collected using the K2EDTA separator had significantly lower BDNF concentrations than the Li-Hep, Li-Hep (gel), and Na-Hep tubes (852.2 ± 155.9 vs. 1867.0 ± 148.7, 1601.0 ± 124.4, 1839.0 ± 148.0; *p* ≤ 0.0001, one-way ANOVA with Tukey’s post hoc), while the Na-Hep (glass) was found to be comparable (1346.0 ± 107.0 pg/mL) (Fig. 2C). Results suggest that the type of plasma separator used affects the quantity of BDNF recovered, especially in women. Since age, sex, and menstrual cycle stage have been shown to affect circulating concentration of BDNF^30,33^, a multivariable linear mixed effects model was employed to evaluate the effect of these covariates on BDNF concentrations for all tubes, at all timepoints, and storage temperatures tested (Supplemental Fig. 1). When all data (for all tubes, storage time, and storage temperature) was combined in our regression analyses, age, sex, and menstrual cycle stage did not significantly affect circulating BDNF concentrations in our cohort. Therefore, subsequent analyses were not stratified by these factors.

### BDNF increases in plasma frozen at − 20 °C for 12 months

**Figure 3:**
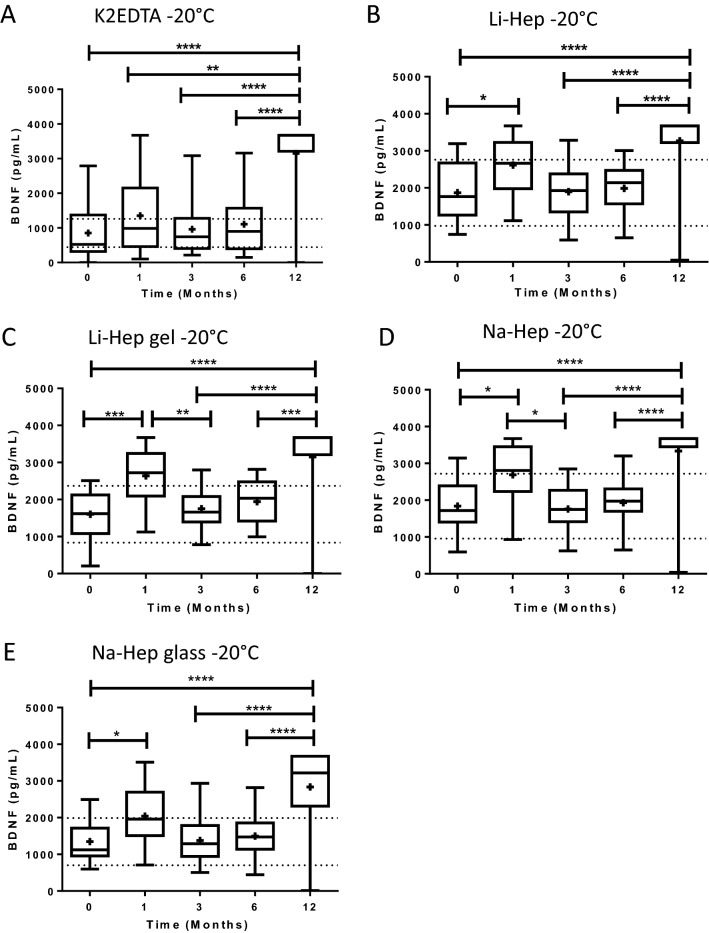
BDNF increases in plasma frozen at − 20 °C for 12 months. (**A**) Plasma isolated using K2EDTA tubes and stored at − 20 °C had increased BDNF by 12 months (*p* =  < 0.0001, Kruskal–Wallis test with Dunn’s post hoc). (**B**) Plasma isolated by lithium-heparin tubes and stored at − 20 °C had increased BDNF by 12 months (*p* ≤ 0.0001, Kruskal–Wallis test with Dunn’s post hoc), and had significant inter-month variability. (**C**) BDNF in plasma isolated from lithium-heparin gel separators increased by 12 months following storage at − 20 °C (*p* ≤ 0.0001, Kruskal–Wallis test with Dunn’s post hoc), and had significant inter-month variability. (**D**) Plasma isolated by sodium-heparin tubes had significantly increased BDNF by 12 months, following storage at − 20 °C (*p* ≤ 0.0001, Kruskal–Wallis test with Dunn’s post hoc), and had significant inter-month variability. (**E**) When plasma was isolated by glass sodium-heparin tube and stored at − 20 °C, BDNF concentration increased significantly by 12 months (*p* ≤ 0.0001, Kruskal–Wallis test with Dunn’s post hoc), and had significant inter-month variability. BDNF: brain-derived neurotrophic factor. K2EDTA: potassium EDTA. Li-Hep: lithium heparin. Na-Hep: sodium heparin. *p ≤ 0.05, **p ≤ 0.01, ***p ≤ 0.001, ****p ≤ 0.0001. Dotted lines indicate the expected range of the means for each plasma separator (calculated to be ± 47.9%, based on inter-assay CV and the mean concentration of BDNF recovered from each plasma separator at Time 0). + : indicates the mean.

The effect of storage time at − 20 °C for up to 12 months on BDNF concentrations was assessed in plasma. The quantity of BDNF in the plasma isolated by the K2EDTA tubes and stored at − 20 °C increased significantly by 12 months, in samples that were only thawed once. The mean quantity of BDNF in the K2EDTA-isolated plasma stored at − 20 °C was 852.2 ± 155.9 at time 0, 1353.0 ± 217.8 at 1 month, 959.7 ± 163.3 at 3 months, 1107.0 ± 162.7 at 6 months, and 3147.0 ± 217.3 pg/mL at 12 months; *p* ≤ 0.0001, Kruskal–Wallis test with Dunn’s post hoc (Fig. 3A). The mean concentrations of BDNF fell within the expected range of the means (± 47.9% of the time 0 mean for each collection tube) at all time points, except 1 (fell just outside of the range) and 12 months post-collection. The increase in BDNF concentration was observed for all storage times versus 12 months, without a step-by-step increase over time (ie. there was no difference between two consecutive timepoints other than 6–12 months). Therefore, the quantity of BDNF recovered from the K2EDTA collected plasma and stored at − 20 °C remained stable up until 6 months.

BDNF quantified in plasma isolated by Li-Hep, Li-Hep (gel), Na-Hep, and Na-Hep (glass) tubes and stored at − 20 °C also increased significantly *p* ≤ 0.0001 (Kruskal–Wallis tests with Dunn’s post hoc) by 12 months (Fig. 3B–E). Furthermore, plasma isolated by these tubes and stored at − 20 °C had more up and down fluctuations at the intermediate timepoints as compared to plasma collected in K2EDTA tubes, and these tubes had a much wider range of acceptable concentrations than the K2EDTA tube (Fig. 3A). Although there was some variability, the mean concentrations of BDNF fell within the expected range of the means (± 47.9% of the time 0 mean) for Li-Hep (Fig. 3B) and Na-Hep (Fig. 3D) at all time points, except 12 months post-collection whereas BDNF concentrations did not fall within the expected range of the means (± 47.9% of the time 0 mean) for the Li-Hep (gel) (Fig. 3C) and Na-Hep (glass) (Fig. 3E) separator tubes at 1 and 12 months post-collection. Due to the fluctuations in BDNF concentrations at intermediate timepoints, it appears as though these tubes (Li-Hep, Li-Hep (gel), Na-Hep, and Na-Hep (glass)) are less likely than K2EDTA to yield an accurate concentration of BDNF for up to 6 months of storage at − 20 °C.

### BDNF increases in plasma frozen at − 80 °C for 12 months

**Figure 4:**
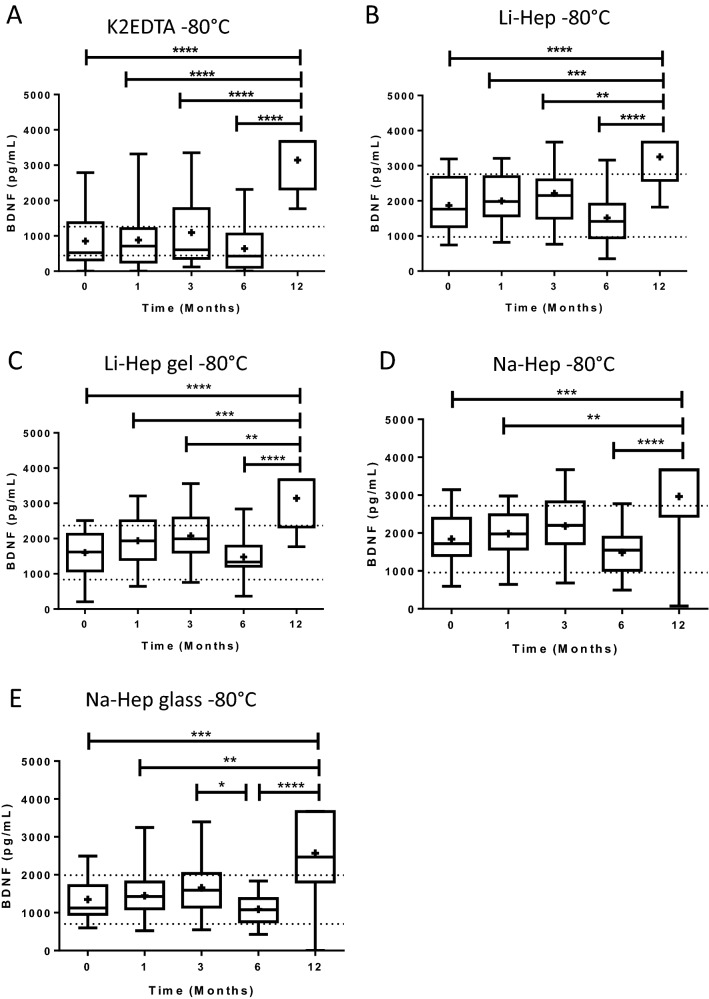
BDNF increases in plasma frozen at − 80 °C for 12 months. (**A**) Plasma collected in K2EDTA tubes and frozen at − 80 °C had significantly increased BDNF by 12 months following collection (*p* ≤ 0.0001, Kruskal–Wallis test with Dunn’s post hoc). The same was observed for (**B**) lithium-heparin tubes (*p* ≤ 0.0001, Kruskal–Wallis), (**C**) lithium-heparin gel separators (*p* ≤ 0.0001, Kruskal–Wallis test with Dunn’s post hoc), (**D**) sodium heparin collection tubes (*p* ≤ 0.0001, Kruskal–Wallis test with Dunn’s post hoc), and (**E**) glass sodium heparin (*p* ≤ 0.0001, Kruskal–Wallis test with Dunn’s post hoc), which was the least stable when plasma isolated by this tube was stored at − 80 °C. Na-Hep (glass) also had significantly different quantities of BDNF recovered at 3 vs. 6 months, and 6 vs. 12 months. BDNF: brain-derived neurotrophic factor. K2EDTA: potassium EDTA. Li-Hep: lithium heparin. Na-Hep: sodium heparin. *p ≤ 0.05, **p <  ≤ 0.01, ***p ≤ 0.001, ****p ≤ 0.0001. Dotted lines indicate the expected range of the means for each plasma separator (calculated to be ± 47.9%, based on inter-assay CV and the mean concentration of BDNF recovered from each plasma separator at Time 0). + : indicates the mean.

The effect of sample storage at − 80 °C for up to 12 months on BDNF concentrations was assessed in plasma. The concentration of BDNF in plasma stored at − 80 °C increased significantly by 12 months post-collection (Kruskal–Wallis tests with Dunn’s post hoc) (Fig. 4). However, unlike plasma stored at − 20 °C, 4 of the 5 collection tubes had stable quantities of BDNF recovered up to 6 months post-collection; there were fewer fluctuations at intermediate timepoints. When plasma collected by the K2EDTA tubes was frozen at − 80 °C, there was a significant increase in BDNF concentration by 12 months following collection (3144.0 ± 141.4 pg/ml) compared to all other time points studied (852.2 ± 155.9 time 0, 880.5 ± 174.7 at 1 month, 1095.0 ± 211.0 at 3 months, and 641.2 ± 131.7 at 6 months; *p* ≤ 0.0001, Kruskal–Wallis test with Dunn’s post hoc) (Fig. 4A). When plasma separated by Li-Hep tubes was stored at − 80 °C, we observed the same phenomenon (1867.0 ± 148.7 time 0, 1997.0 ± 151.0 at 1 month, 2215.0 ± 170.5 at 3 months, 1514.0 ± 139.8 at 6 months, and 3250.0 ± 135.0 pg/mL at 12 months; *p* ≤ 0.0001, Kruskal–Wallis test with Dunn’s post hoc) (Fig. 4B).

BDNF concentration also increased by 12 months in plasma isolated by Li-Hep (gel) separators and stored at − 80 °C (1601.0 ± 124.4 time 0, 1938.0 ± 136.1 at 1 month, 2075.0 ± 136.1 at 3 months, 1476.0 ± 112.9 at 6 months, and 3144.0 ± 141.4 pg/mL at 12 months; *p* ≤ 0.0001, Kruskal–Wallis test with Dunn’s post hoc) (Fig. 4C). The same trend was observed for plasma obtained using the Na-Hep collection tubes and stored at − 80 °C (1839.0 ± 148.0 time 0, 1979.0 ± 118.4 at 1 month, 2185.0 ± 168.6 at 3 months, 1483.0 ± 119.2 at 6 months, and 2963.0 ± 222.3 pg/mL at 12 months; *p* ≤ 0.0001, Kruskal–Wallis test with Dunn’s post hoc) (Fig. 4D). Finally, plasma collected using the Na-Hep (glass) was the least stable when stored at − 80 °C. The concentration of BDNF increased over time (1346.0 ± 107.0 time 0, 1450.0 ± 121.1 at one month, 1657.0 ± 128.3 at 3 months, 1089.0 ± 83.98 at 6 months, and 2567.0 ± 214.5 pg/mL at 12 months; *p* ≤ 0.0001, Kruskal–Wallis test with Dunn’s post hoc) (Fig. 4E). However, this collection tube was the only tube unable to maintain consistent BDNF quantities up to 6 months post-collection (Fig. 4E, 3 months vs. 6 months) even though the mean concentrations of BDNF fell within the expected range (± 47.9% of the time 0 mean for each collection tube). Taken together, the quantity of BDNF remained stable in plasma stored at − 80 °C for up to 6 months when isolated using any of the plasma separator tubes, and the mean concentration of BDNF fell within the expected range of the means (± 47.9% of the time 0 mean for each collection tube) at all time points, except 12 months post-collection, suggesting that plasma stored at − 80 °C is more stable than plasma stored at − 20 °C.

### Plasma from K2EDTA tubes stored at − 20 °C or − 80 °C is optimal for BDNF quantification up to 6 months

**Figure 5:**
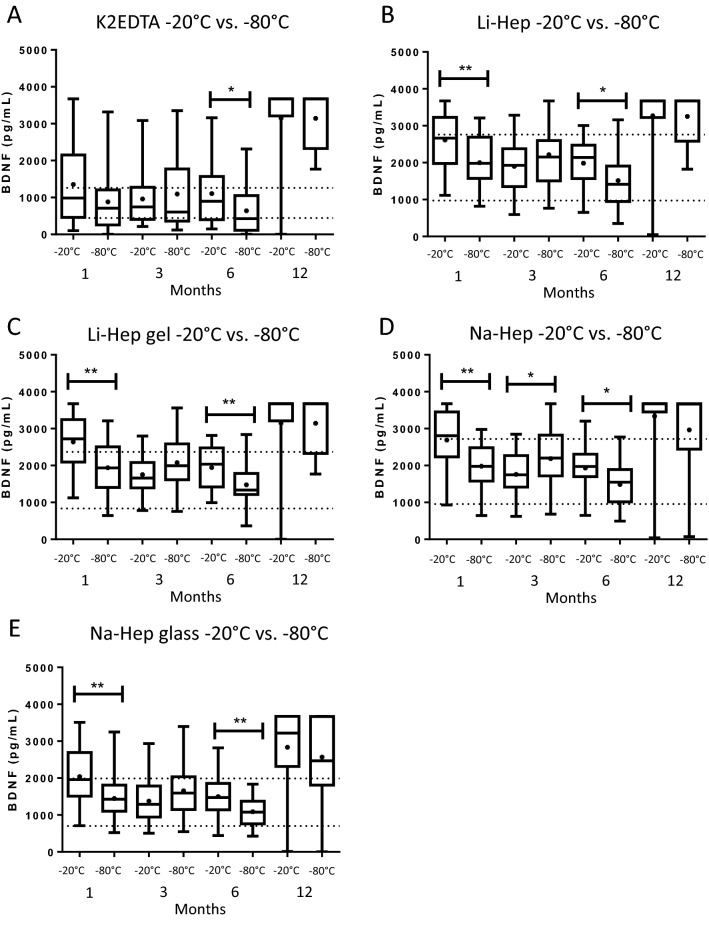
Plasma from K2EDTA tubes stored at − 20 or − 80 °C is optimal for BDNF quantification up to 6 months. The quantities of BDNF recovered from plasma stored at − 20 versus − 80 °C were compared by t-test or Mann–Whitney at 1, 3, 6, and 12 months following collection to determine which tube had the least amount of variability over time and temperature. (**A**) The concentration of BDNF remained stable in plasma collected using K2EDTA tubes, except for at 6 months where there was a significant difference in the BDNF recovered in plasma stored at − 20 versus − 80 °C (*p* = 0.02, Mann–Whitney). (**B**) BDNF recovered from plasma collected by lithium heparin (*p* = 0.007, at 1 month; *p* = 0.02, at 6 months; t-tests), and (**C**) lithium heparin gel (*p* = 0.002, at 1 month; *p* = 0.007, at 6 months; t-tests) varied by storage temperature at 1, and 6 months post-collection. (**D**) The sodium heparin tube was the least stable because the quantity of BDNF recovered and stored at − 20 °C versus − 80 °C varied significantly at 1, 3, and 6 months post-collection (*p* = 0.001, at 1 month; *p* = 0.05, at 3 months; *p* = 0.02, at 6 months; t-tests). (**E**) The sodium heparin glass (*p* = 0.005, Mann–Whitney at 1 month; *p* = 0.008, t-test at 6 months) also varied by storage temperature at 1, and 6 months post-collection. BDNF: brain-derived neurotrophic factor. K2EDTA: potassium EDTA. Li-Hep: lithium heparin. Na-Hep: sodium heparin. *p ≤ 0.05, **p ≤ 0.01. Dotted lines indicate the expected range of the means for each plasma separator (calculated to be ± 47.9%, based on inter-assay CV and the mean concentration of BDNF recovered from each plasma separator at Time 0). + : indicates the mean.

The quantities of BDNF quantified in plasma stored at − 20 °C versus − 80 °C were compared by t-test or Mann–Whitney at 1, 3, 6, and 12 months following collection (Fig. 5) to determine which tube had the least amount of variability over time and temperature. The concentration of BDNF remained stable in plasma collected using K2EDTA tubes, except for at 6 months where there was a significant difference in the BDNF recovered in plasma stored at − 20 versus − 80 °C (1107.0 ± 162.7 vs. 641.2 ± 131.7 pg/mL; *p* = 0.02, Mann–Whitney) (Fig. 5A). In spite of this variability, the mean concentration of BDNF consistently fell within the expected range of the means (± 47.9% of the time 0 mean for each collection tube) for both temperatures and all time points up to 6 months post-collection except for − 20 °C stored for 1 month.

The plasma collected by Li-Hep (2616.0 ± 159.2 vs. 1997.0 pg/mL at 1 month; *p* = 0.007; 1986.0 ± 138.7 vs. 1514.0 ± 139.8 pg/mL at 6 months; *p* = 0.02, t-tests) (Fig. 5B), and Li-Hep (gel) (2641.0 ± 157.0 vs. 1938.0 ± 136.1 pg/mL at 1 month; *p* = 0.002; 1942.0 ± 121.9 vs. 1476.0 ± 112.9 pg/mL at 6 months; *p* = 0.007, t-tests) (Fig. 5C) tubes had BDNF values that varied by storage temperature (− 20 °C vs. − 80 °C) at 1, and 6 months post-collection. The Na-Hep tube was the least stable when comparing storage at − 20 °C versus − 80 °C because the quantity of BDNF recovered varied significantly at 1, 3, and 6 months post-collection (2688.0 ± 171.0 vs. 1979.0 ± 118.4 pg/mL at 1 month; *p* = 0.001; 1760.0 ± 123.9 vs. 2185.0 ± 168.6 pg/mL at 3 months; *p* = 0.05; 1932.0 ± 132.9 vs. 1483.0 ± 119.2 pg/mL at 6 months; *p* = 0.02, t-tests) (Fig. 5D). Finally, the BDNF recovered by the Na-Hep (glass) tube (2041.0 ± 158.2 vs. 1450.0 ± 121.1 pg/mL at 1 month; *p* = 0.005, Mann–Whitney; 1497.0 ± 117.7 vs. 1089.0 ± 83.98 pg/mL at 6 months; *p* = 0.008, t-test) (Fig. 5E) also varied by storage temperature at 1, and 6 months post-collection.

Since there were the fewest significant differences in the quantity of BDNF recovered from plasma collected in the K2EDTA tubes and stored at − 20 versus − 80 °C, we suggest this is the optimal tube to use for collection. Storage temperature (− 20 vs. − 80 °C) for up to 6 months in the freezer did not affect the BDNF concentration measured in K2EDTA tubes (mean BDNF concentrations generally fell within our calculated acceptable change limit up to 6 months in the freezer). We therefore suggest that plasma collected in the K2EDTA tubes and stored at − 20 °C or − 80 °C for up to 6 months represent the optimal conditions under which to recover and accurately quantify circulating BDNF.

### BDNF increases following freeze–thaw cycles

**Figure 6:**
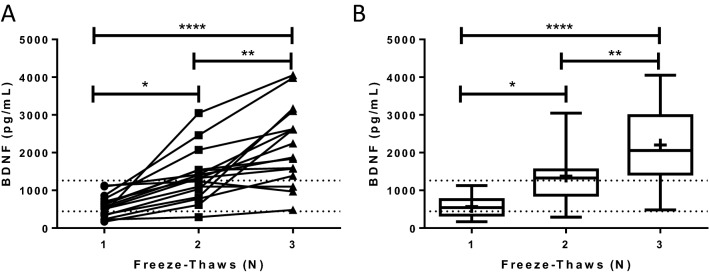
BDNF increases following freeze–thaw cycles. (**A**) The quantity of BDNF in the plasma collected in K2EDTA tubes from a prior study increased in women, over repeated freeze-thaws. The values obtained from the plasma of each woman after each freeze–thaw cycle are connected by the lines in the plot. (**B**) The mean quantity of BDNF significantly increased as the number of repeated freeze–thaw cycles increased (*p* ≤ 0.0001, one-way ANOVA with Tukey’s post hoc). BDNF: brain-derived neurotrophic factor. *p ≤ 0.05, **p ≤ 0.01, ****p ≤ 0.0001. Data in (**A**) are presented as individual plasma samples, while data in (**B**) are presented as a box plot (25th, 50th (median), 75th percentiles). Dotted lines indicate the expected range of the means for the K2EDTA plasma separator (calculated to be ± 47.9%, based on inter-assay CV and the mean concentration of BDNF recovered from each plasma separator at Time 0). + : indicates the mean.

The effect of repeated freeze–thaw cycles was tested using aliquots of plasma isolated between April 2011 and December 2012 using K2EDTA plasma separator tubes obtained during our prior study^20^. The quantity of BDNF in the plasma was significantly (*p* ≤ 0.0001) higher over repeated freeze–thaw cycles (Fig. 6A). The amount of BDNF significantly increased as the number of repeated freeze–thaw cycles increased (572.6 ± 7 2.68 vs. 1376.0 ± 172.7 vs. 2200.0 ± 260.2 pg/mL for 1, 2, and 3 freeze-thaws respectively; *p* ≤ 0.0001, one-way ANOVA with Tukey’s post hoc) (Fig. 6B).

## Discussion

Our results demonstrate that the type of plasma separator tube, storage duration, and number of freeze–thaw cycles are important factors affecting measurement of plasma BDNF concentrations. We also demonstrate that freezer storage temperature (− 20 ºC vs. − 80 ºC) does not significantly impact the amount of BDNF recovered from frozen plasma, although plasma stored at − 80 °C tended to have less variability in mean BDNF concentrations between time points. In contrast, storage of samples beyond 6 months resulted in a significant increase in plasma BDNF concentrations, for all tubes assessed in this study. Taken together, our results suggest that K2EDTA is the preferred anticoagulant and that storage for up to 6 months at either − 20 or − 80 °C produces reproducible results when quantifying circulating BDNF. However, repeated freeze-thaws should be avoided for plasma isolated using K2EDTA, as this significantly affects the quantity of BDNF measured.

In the present study, there was no overall effect of sex, age, or menstrual cycle stage on the concentration of BDNF when we performed regression analyses on all BDNF data combined (all tubes, all temperatures, all study participants). However, we did find that when we looked at only plasma BDNF concentrations measured in samples collected using K2EDTA separator tubes, that the concentration of BDNF was significantly lower in the K2EDTA tube than the other anticoagulant tubes for females but not for males. When we combined the data from males and females, the plasma BDNF concentration measured in fresh plasma samples from healthy volunteers (combined, Fig. 2C) was significantly lower when the anticoagulant K2EDTA was used compared to all other anticoagulants except for Na-Hep (glass). As BDNF is stored in platelets^26–28^, we speculate that the K2EDTA tube likely provides the most accurate quantification of free BDNF in the plasma, while the other anticoagulants tested may have resulted in a greater degree of platelet degranulation. Prior studies have not compared the effect of type of plasma separator tube used, although the BDNF concentration was higher than what we observed in our study when samples were collected in K2EDTA and stored at 25 °C before analysis by ELISA in another study^38^. Our results also demonstrated that the plasma concentration of BDNF remained stable over time when frozen at either − 20 or − 80 °C up to 6 months, beyond which the concentration of BDNF measured in the samples increased for all anticoagulants used. We speculate that the increased concentration of BDNF following 12 months in the freezer is most likely the consequence of partial or complete degranulation of residual platelets retained in the plasma sample. Our results are consistent with and expand on previous findings for samples collected with EDTA and stored at − 80 °C for 6 months^39^. Taken together, we suggest that for reproducible results when quantifying BDNF in plasma, frozen samples stored for BDNF analysis should be analyzed before 6 months.

In the present study, concentrations of BDNF increased with multiple freeze–thaw cycles, in plasma isolated using K2EDTA tubes. These results were unexpected relative to other proteins such as parathyroid hormone^40^ and anti-mullerian hormone^41^ which decline with repeated cycles of freezing and thawing, whereas others are reported to remain unchanged^40^. Our results also contrast with a prior report in which plasma BDNF concentrations remained within the acceptable range of measurement over two-cycles of freezing and thawing^39^. However, consistent with our data, there was an increase in both the plasma and serum concentrations of BDNF in their report. We suggest that the most likely explanation for the increase in plasma BDNF concentrations with repeated freeze–thaw cycles is that commercial plasma separation tubes cannot eliminate all platelets from the sample and residual platelets in the plasma release BDNF into the sample upon repeated freezing and thawing.

Since the type of plasma separator tube (anticoagulant), storage time, and freeze–thaw cycles were found to influence the amount of BDNF recovered from plasma, we suggest caution should be taken in future studies aimed at supporting or refuting BDNF as a useful clinical marker of disease. Further, the methods and storage conditions used should be assessed prior to attempting to compare results between studies. Taken together, we suggest that K2EDTA plasma separator tubes are preferred for BDNF measurement owing to stability in BDNF measures over 6 months of storage at both − 20 and − 80 °C. Moreover, BDNF concentrations measured in samples collected using K2EDTA tubes demonstrated a narrower expected range of concentrations compared to the other plasma separator tubes used.

This study had a number of strengths as well as limitations that merit discussion. The prospective study design using healthy volunteers of both sexes was considered a strength of the study. In addition, collection of all blood samples on a single day over a narrow time window together with storage of samples on ice immediately after the blood draw and for transport to the laboratory is thought to have minimized the influence of diurnal variation and the initial impact of platelet degranulation. All samples were processed together by the same technician and within approximately 4 h of sample collection. All samples for each time point were analyzed together to minimize variation from the use of assay kits from different lot numbers. There are also a number of important weaknesses with the present study. For example, study subjects were not asked to fast before providing a sample and we were unable to control for participant body mass index. Additionally, the ELISA kit used quantified both mature and proBDNF, and although these isoforms can be separately measured in serum^36^, we are unable to distinguish whether the effects seen in our study mainly affect mature BDNF, proBDNF, or both isoforms equally. Furthermore, we did not assess the potential impact of hemolysis within the plasma, which we observed in a few plasma samples. It is possible that these factors also affect the quantity of BDNF recovered from plasma. Furthermore, multiple linear regressions on our data did not find an effect of participant age, sex, and menstrual cycle stage on circulating BDNF concentrations (for all tube types, at all timepoints, and storage temperatures), even though these factors have previously been shown to influence BDNF^30,33^. Only the K2EDTA tube stored at − 80 °C for 12 months showed an effect, where age was significantly associated with the concentration of BDNF (*p* = 0.038, multiple linear regression). We suspect we were not able to replicate the effect of age, sex, and menstrual cycle stage on concentration of BDNF in our sample, due to our small sample size, and narrow age range. Finally, although it is well established that platelets are the major source of peripheral BDNF^6,30,42,43^, we did not quantify platelet numbers in the samples at the time of collection. Measuring residual platelet counts in plasma samples could provide insight into changes in BDNF concentrations over time.

## Conclusions

While all of the anticoagulant plasma separator tubes we tested produce reliable results, we suggest that the K2EDTA separator tube is preferable for the quantification of free BDNF in plasma because it appeared to give the most stable quantification of BDNF for up to 6 months of storage at − 20 and − 80 °C (least variability between intermediate time points). Furthermore, storage temperature (− 20 °C vs. − 80 °C) had no effect on plasma BDNF concentrations which remained stable up to 6 months in storage in all five anticoagulant systems used. However, we suggest consistency in sample collection (including type of plasma separator used), processing, and storage (− 80 °C preferred because the mean concentration of BDNF fell within the expected range of the means at all time points, except 12 months post-collection) because the quantification of free BDNF presents unique challenges, given that it is stored in platelets. Furthermore, the storage of samples beyond 6 months and repeated freeze–thaw cycles should be avoided, because these factors also affect the concentration of BDNF recovered from plasma.

Taken together, we suggest that plasma collected in the K2EDTA tubes and stored at − 20 °C or − 80 °C for up to 6 months and only thawed once represent the optimal conditions under which to accurately quantify circulating BDNF.

## Supplementary information

Supplementary Figure 1.

Supplementary Tables.

## Data Availability

Supporting raw data is available in Supplementary Table 1.
