# Supplementary material for: Factors affecting stability of plasma brain-derived neurotrophic factor
Source: Sci Rep. 2020 Nov 19;10:20232. doi: 10.1038/s41598-020-77046-6 (PMC7677545; doi:10.1038/s41598-020-77046-6)
Supplement: Supplementary file 1 — Supplementary Figure 1. [file 41598_2020_77046_MOESM1_ESM.docx]

**Factors Affecting Stability of Plasma Brain-derived Neurotrophic Factor**

Jocelyn M. Wessels^1¶^, Ravi K. Agarwal^2¶^, Aamer Somani^1^, Chris P. Verschoor^3^, Sanjay K. Agarwal^2^, and Warren G. Foster^1,2*^

**^1^**Department of Obstetrics and Gynecology, McMaster University, Hamilton, ON, Canada

^2^Department of Obstetrics & Gynecology and Reproductive Science, University of California, San Diego, La Jolla, California, USA

^3^Health Sciences North Research Institute, Sudbury, ON, Canada

***^¶^Joint first authors***

^*^Corresponding author: Dr. Warren G. Foster, Ph.D.

Department of Obstetrics & Gynecology, HSC-3N52D

McMaster University,

1280 Main Street, West,

Hamilton, Ontario, Canada

L8S 4K1

Email: [fosterw@mcmaster.ca](mailto:fosterw@mcmaster.ca)

***Running Title:*** Sample processing and plasma BDNF stability

***Keywords:*** BDNF, anticoagulant, temperature, time, vacutainer, storage, freeze, thaw, ELISA, endometriosis.

# Supplemental information

**Supplemental Figure 1. Association of participant demographics with BDNF concentration across different testing conditions.** BDNF concentration was regressed on tube type (reference: K2EDTA), time (reference: time 0), and freezer temperature (reference: -20 °C), in addition to (A) age and sex, or (B) age and menstrual cycle stage (reference: hormonal contraceptives). For A, all participants were included, and for B, only female participants were included. The regression coefficient (β) and 95% confidence interval are presented on the x-axis and parameters were considered statistically significant if the 95% confidence interval did not cross 0 (no difference). K2EDTA: potassium EDTA. Li-Hep: lithium heparin. Na-Hep: sodium heparin.
